# Integrating *in vitro* digestion and peptidomics into novel food allergenicity assessment: A study on *Clostridium tyrobutyricum* biomass

**DOI:** 10.1016/j.crfs.2025.101237

**Published:** 2025-11-03

**Authors:** Vaios D. Fytsilis, Rensong Ji, Karli R. Reiding, Albert J.R. Heck, Frederik-Jan van Schooten, Alie de Boer, Misha F. Vrolijk

**Affiliations:** a Department of Pharmacology and Toxicology, Nutrition and Translational Research Institute in Metabolism – NUTRIM, Faculty of Health, Medicine and Life Sciences, Maastricht University, Maastricht, the Netherlands; b Biomolecular Mass Spectrometry and Proteomics, Bijvoet Center for Biomolecular Research and Utrecht Institute for Pharmaceutical Sciences, Utrecht University, Utrecht, the Netherlands; c Netherlands Proteomic Center, Utrecht, the Netherlands; d Food Claims Research Centre, Faculty of Science and Engineering, Maastricht University, Venlo, the Netherlands

## Abstract

Assessing the allergenicity of novel foods (NFs) is a complicated process that requires data on multiple product aspects. Although the link between allergenicity and protein digestibility remains debated, integrating next-generation risk assessment (NGRA) tools into this process is increasingly recognized as essential. Here, we present an integrated approach combining *in vitro* digestion, peptidomics, and *in silico* analysis to evaluate the allergenic potential of a complex NF mixture.

*Clostridium tyrobutyricum* biomass was digested *in vitro*, and samples collected at multiple digestion time points were analyzed by SDS-PAGE and LC-MS/MS-based proteomics. Generated peptides were filtered using allergenicity-relevant criteria and examined for sequence, size, persistence, and abundance. Proteins were further screened with online tools to predict cross-reactivity with known allergens, and resulting peptides were analyzed to determine whether potential epitopes persisted after digestion.

This workflow presents a flexible and efficient methodology to assess multiple allergenicity-relevant endpoints from a single dataset, offering a valuable screening step within current risk assessment frameworks. By integrating complementary experimental and computational tools, the method enables a more holistic and mechanistic understanding of allergenic potential in complex mixtures. Overall, the current work demonstrates how peptide persistence and abundance can be meaningfully integrated with *in silico* predictions to better estimate allergenic potential, while outlining current limitations and uncertainties in NGRA-based allergenicity assessment.

## Highlights

• There is a need for the integration of advanced tools into novel food risk assessment.
• Peptidomics offer great insight into the effect of *in vitro* digestion on proteins.
• Allergenic potential can be assessed based on generated peptides.
• Integration of this approach could complement the current regulatory framework.

## Graphical abstract

**Figure:**
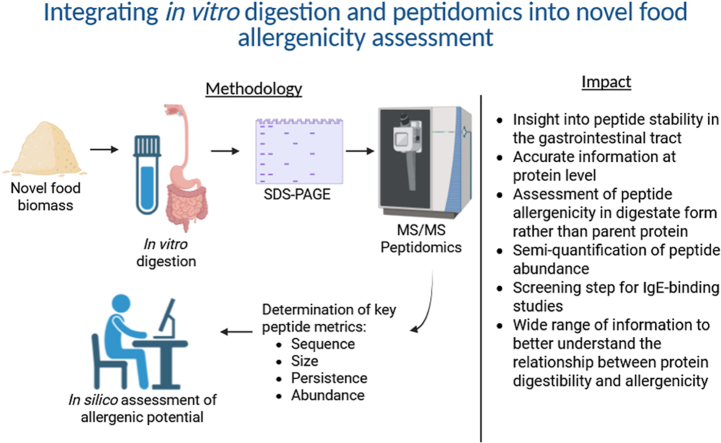

## 1 Introduction

Novel foods (NFs) are defined in the European Union (EU) as foods that had not been consumed to a significant degree by humans in the EU before the May 15, 1997 (Regulation 2015/2283). Before such products can be placed on the EU market, they need to undergo a rigorous safety review and subsequent authorisation by the European Commission. Since 2018 there has been a substantial increase in NF applications received by the European Food Safety Authority (EFSA) relating to NFs derived from microorganisms, fungi or algae (Ververis et al., 2020). Microbial biomass produced for food consumption is often referred to as single-cell protein (SCP) and it has received a great deal of interest from the food industry in the past few years as a sustainable way of food production (Nyyssölä et al., 2022). Finnish startup Solar Foods (solarfoods.com), which makes “food out of thin air” utilizing gas-fed bacterial culture, Superbrewed Food (superbrewedfood.com) and their postbiotic cultured protein, also produced from bacteria, and The Protein Brewery (theproteinbrewery.nl) that produces fungal-based “Fermotein” are only a few examples of companies trying to create sustainable protein-rich foods through microbial fermentation. All three of these ingredients have completed various regulatory pathways in the United States or Singapore, while all three have submitted novel food applications and are undergoing evaluation by the EFSA, the agency responsible for food risk assessment in the EU (de Boer and Bast, 2018).

The health risks associated with the use of SCPs in food, along with the regulatory requirements that they will need to meet (in the EU) have been extensively covered by us and others (Ritala et al., 2017; Banach et al., 2023; Fytsilis et al., 2024). The current strategy focuses specifically on the potential allergenicity from the consumption of novel microorganisms. These novel biomasses remain somewhat an “uncharted territory” in risk assessment, as they present daunting challenges regarding both their origin and nature as complex mixtures that might necessitate more extensive analyses (Turck et al., 2024). In their quest to maximize production efficiency, companies can experiment with various species of unicellular microorganisms, many of which may not be well characterized in terms of allergenic potential or have phylogenetic relations that remain unknown. The current EFSA guidelines for allergenicity assessment of novel complex protein mixtures follow a tiered approach that begins with a comprehensive literature review and a phylogenetic analysis of the source (micro)organism to establish potential relationships with known allergenic sources (Tier I) (Turck et al., 2024). This study can, on a case-by-case basis, be followed by bioinformatics analysis (Tier II), exploring the homology between known allergens and proteins of interest and even specific human serum IgE-binding (Tier III) or human (Tier IV) studies (e.g., skin-prick tests) in case of strong evidence of cross-reactivity. Although phylogenetic and sequence homology studies remain an extremely useful and dependable tool in allergenicity assessment, they often depend on the presence of related organisms and/or allergens in foods, and, logically, become less reliable for more distant phylogenetic relationships (Weber, 2003; Turck et al., 2024; Mills et al., 2024). Moreover, these studies do not offer the accuracy of mass-spectrometric protein identification, neither do they account for the behavior of proteins and other food components in the human gastrointestinal (GI) tract. Therefore, in our assessment of the allergenic potential of a known bacterial SCP we employ a combination of *in vitro* digestion and peptidomics methods, aimed at developing a refined screening strategy that can identify proteins of potential concern for IgE-mediated allergenicity assessment from complex food mixtures. This paper does not aim to propose a new allergenicity assessment strategy altogether. Instead, it explores how advanced next generation risk assessment (NGRA) tools can enhance the current framework and help investigate obscure mechanisms, such as the one that connects allergen stability in the GI tract to allergenic potential.

## 2 Materials and methods

### 2.1 Sample

The SCP test sample was bacterial biomass of the species *Clostridium tyrobutyricum*, commercialized and provided by Superbrewed Food (Delaware, United States). After anaerobic fermentation the biomass of dried killed bacterial cells was heat treated to reduce nucleic acid content and was received in powder form without any further processing, as intended to be marketed. The powder has an estimated total protein content of around 85 % (w/w) and less than 3 % crude fat (Jonaitis et al., 2022). *Clostridium tyrobutyricum* is a gram-positive bacterium, often used in cheese making, described on the EFSA Qualified Presumption of Safety (QPS) list since December of 2023. In the form of dried biomass, it has been extensively studied regarding its safety as a NF (Jonaitis et al., 2022) and received a “No-Questions” letter from the United States Food and Drug Administration in March of 2024.

### 2.2 *In vitro* digestion and digested sample generation

Digestion of the sample was performed *in vitro* according to the protocol by Brodkorb et al. (2019) with minor adjustments, using a starting concentration of 50 mg/mL which corresponds to a total protein concentration of 41.5 mg/mL. The powder was dispersed in water and shaken briefly to imitate the texture and protein content of a commercial protein drink or shake. Due to the negligible starch content of the sample, the use of human salivary amylase in the oral phase was omitted. Additionally, the use of rabbit gastric lipase, which is recommended in the protocol, was also excluded from the digestive process, therefore the methodology does not account for gastric lipid digestion and any resulting lipid-protein interactions that may affect peptide persistence in the stomach. This omission was deemed acceptable, due to the very low (<0.2 % w/v) fat content of the starting meal (Jonaitis et al., 2022) and the fact that rabbit gastric lipase of high purity was not commercially available in the few months prior to the experiments. Faced with the choice of using lower purity gastric lipase and consequently replacing a significant part of the porcine pepsin with rabbit pepsin and considering the low fat content of the product, we decided to exclude the use of gastric lipase altogether, as rabbit pepsin shows much lower homology to human pepsin than the porcine enzyme (BLAST) (Altschul et al., 1990), and our main objective was achieving protein digestion in human-like manner.

Before the digestion experiments, enzyme activities (and bile acid concentration) were determined in multiple (≥3) technical replicates and the mean value was taken as final activity, and a pH adjustment experiment was run for the sample, all according to the protocol recommendations. Test samples of 1 mL were obtained at three different digestion timepoints (i.e., 2,3 and 4 h of total digestion), with each timepoint being represented by a respective digestion tube. After 2 h of gastric digestion with pepsin (P7012, Sigma Aldrich), samples were collected and the pH was immediately adjusted to the range of 7–7.5 by addition of sodium hydroxide solution (1M) to achieve pepsin denaturation. In the intestinal phase, bovine bile (B3883, Sigma Aldrich) was incorporated, and trypsin and chymotrypsin were added in the form of porcine pancreatin (P7545, Sigma Aldrich). Samples were obtained after one and 2 h of intestinal digestion and were immediately mixed with Bowman-Birk protease inhibitor (T9777, Sigma Aldrich). Furthermore, control tubes containing only enzymes and no food (blank) and only food without enzymes (stability) were run and samples were obtained at the end of digestion, with blank samples also being mixed with inhibitor. All digested samples from all timepoints and phases were snap-frozen in liquid nitrogen immediately after collection and stored at −80^o^C until further analysis was needed. The digestion protocol was run in triplicate for each test and control tube.

### 2.3 SDS-PAGE analysis

Gel electrophoresis was performed according to the protocol of the gel manufacturer (Bio-Rad), with adjustments. Digestion samples were thawed at room temperature and diluted with Milli-Q ultrapure water to an estimated food protein content of 3 mg/mL (based on starting food protein content) and then mixed 1:1 with 2X non-reducing sample buffer (62.5 mM Tris-HCl, pH 6.8; 25 % glycerol; 2 % SDS; 0.01 % bromophenol blue). Samples were then heated for 10 min at 70^o^C, to achieve protein structure denaturation, spun down for 5 min and briefly let cool down at room temperature. Using a gel loader tip, 10 μL of each sample and Precision Plus Protein™ Unstained Protein Standards (Bio-Rad) were loaded into a 4–20 % Mini-PROTEAN® TGX™ Precast Protein Gel. After loading, electrophoresis was run at 90V for 5 min and at 125V until the dye front reached the designated cassette line (35–40 min). Immediately after, gels were placed in 50 mL of fixing solution (40 % ethanol, 10 % acetic acid) on a rocking table for 1 h, followed by momentary washing with Milli-Q water and staining with 100 mL Bio-Safe™ Coomassie Stain for 1 h. Finally, gels were destained in 250 mL Milli-Q water overnight and imaged at high resolution using a ChemiDoc Imaging System (Bio-Rad). Gel electrophoresis was performed for samples from all three digestion runs to ensure reproducibility of the findings.

The selection of experimental conditions (e.g., non-reducing buffer, gel porosity) was due to the perceived nature of the sample. NFs usually comprise complex protein mixtures and their behavior during digestion and analysis cannot be predicted, as could be the case with purified proteins. Therefore, chemical treatment was kept at the minimum while obtaining as wide a collection of information as possible, as if we were working with unknown food samples.

### 2.4 LC-MS/MS analysis

Samples were prepared as described in Portmann et al. (2023) with modifications. Samples were thawed on ice and vortexed, after which 25 % of each sample was aliquoted for further processing. Intact proteins were removed by adjusting the solution to 80 % acetonitrile with 0.1 % formic acid, followed by incubation on ice for 1 h. The samples were then centrifuged at 15,000×*g* for 15 min at 4 °C. The resulting protein pellet was discarded, and the peptide-containing supernatant was collected. Acetonitrile was evaporated using vacuum centrifugation, and the samples were reconstituted in 2 % formic acid for further cleanup. Peptide desalting was performed using C18 solid-phase extraction columns (Sep-Pak, Waters), followed by drying via vacuum centrifugation. The final samples were reconstituted in 0.1 % trifluoroacetic acid, and 0.5 % of each sample was analyzed by liquid chromatography tandem mass spectrometry (LC-MS/MS).

Chromatographic separation was performed using a two-buffer system, where Buffer A consisted of water with 0.1 % formic acid, and Buffer B contained 80 % acetonitrile and 20 % water with 0.1 % formic acid. Peptides were initially trapped for 1 min at a flow rate of 30 μL/min with 100 % Buffer A on a trap column (0.3 mm × 5 mm, PepMap C18,5 μm, 100 Å; Thermo Fisher Scientific). Following trapping, peptides were separated using a 50-cm analytical column packed in-house with C18 beads (ReproSil-Pur 120 C18-AQ, 1.9 μm; Dr. Maisch). The chromatographic gradient consisted of an increase from 4 % to 44 % Buffer B over 39 min at a flow rate of 300 nL/min, followed by an increase to 55 % Buffer B in 4 min and a final step to 99 % for column cleaning.

MS was performed on an Orbitrap Exploris 480 system (Thermo Fisher Scientific, USA). For analysis, peptides were ionized using a spray voltage of 2 kV, with a capillary temperature of 275 °C. Full-scan MS spectra were acquired in the mass range of m/z 375–1600 with a resolution of 60,000. The maximum injection time was set to Auto, and the automated gain control (AGC) target was set to Standard. Dynamic exclusion was applied for 10 s within an exclusion window of 10 ppm, with a cycle time of 1 s. Charge-state screening was enabled, and precursor ions with charge states of 2+ to 6+ and intensities greater than 2 × 10^5^ were selected for MS/MS analysis. Fragmentation was performed using higher-energy collisional dissociation (HCD), with MS/MS spectra acquired in the Orbitrap mass analyzer at a resolution of 15,000 (isolation window of 1.4 Th), an AGC target set to Standard, and a normalized collision energy (NCE) of 28 %.

### 2.5 Data analysis

Raw data files were analyzed using MaxQuant (version 2.6.6.0) with default settings, except where specified. Deamidation of asparagine (N) was included as a variable modification, and the digestion mode was set to unspecific. The maximum peptide length for unspecific search was set to 80 amino acids (aa). The reference proteome (UP000019482), comprising 3032 protein sequences, was obtained from UniProt in October 2023. To refine the search space, the protein sequences of pig pancreatic amylase (P00690), pig trypsin (P00761), pig pepsin (P00791), pig colipase 1 (P20261), colipase 2 (P32946), colipase 3 (P32947), and bovine chymotrypsin (P00766) were excluded from the list of contaminants and analyzed alongside the reference proteome. Peptidomics analysis was conducted using the peptides.txt file from the MaxQuant output. Peptides derived from contaminants and reversed sequences were removed. Only sequences with MaxQuant scores >60 and PEP score ≤0.1 were retained for further analysis.

Randomized triplicates were analyzed for each sample and timepoint. Pepsin is a proteolytic enzyme of low specificity, and its activity can lead to generation of various peptides from the lysis of the same protein, all of which could theoretically be relevant when assessing the overall allergenic potential. Nonetheless, for demonstrative purposes and to avoid graphs and tables with thousands of entries, only peptide fragments appearing in all three replicates were retained. Furthermore, peptides introduced by the addition of digestive enzymes were filtered out. For every unique sequence the mean intensity was obtained from each set of triplicates and then the mean values were normalized and expressed as z-score. Normalization allows for the semi-quantitative expression of peptide abundance in the sample based on relative intensity (terms used interchangeably from now on).

Formula for calculating z-score:

$$z=\frac{x-\mu }{\sigma }$$

**x** = peptide intensity mean, **μ** = sample intensity mean, **σ** = sample intensity standard deviation.

Peptides longer than or equal to 9aa in length were retained and evaluated for persistence between digestive phases. The cutoff value of 9aa was selected as it suggested by EFSA guidance to be the lowest peptide length that is likely to generate an immune response, as shorter peptides are probably unable to bind to MHC class II molecules (Naegeli et al., 2017; Mohan and Unanue, 2012). Large peptides, defined as peptides of mass larger than or equal to 2.45 kDa were further evaluated for persistence and abundance in different phases. Selecting a cutoff value for IgE cross-reactivity screening proved to be more challenging as there is no clear consensus on a length or mass value below which peptides can be considered non-reactive. The EFSA guidance acknowledges the potential role of peptide aggregation as well as the need for a minimum of two distinct epitopes to trigger degranulation and recommends a peptide mass value between 3 and 5 kDa as the necessary minimum to trigger cross-linking of IgE antibodies bound to the surface of effector cells (Naegeli et al., 2017). However, in one of the latest EFSA-funded scientific reports citing more recent studies, the peptide mass cutoff value for IgE cross-reactivity appears to be 2.5 kDa (Mills et al., 2024), while in the most recent EFSA GMO Panel scientific opinion the cutoff is suggested to be approximately 3 kDa (Casacuberta et al., 2025). Therefore, to broaden the peptide inclusion criteria for this study as much as reasonably possible, we have selected 2.45 kDa as the peptide mass cutoff value for cross-reactivity searches. For large peptides, the parent protein sequences were obtained in FASTA format and run against two known allergen databases, AllergenOnline (Goodman et al., 2016) and AllerCatPro 2.0 (Nguyen et al., 2022) to determine potential sequence identity of 35 % or higher over an 80aa window, and the union of the resulting sets of positive hits from both databases was acquired (Pearson and Lipman, 1988). The choice of database can impact the result of the assessment. AllergenOnline can only handle one sequence at a time, but it gives information on multiple matches, while AllerCatPro 2.0 also gives information on multiple matches but can handle FASTA files of multiple sequences at a time, which can be very useful for high-throughput analyses. Finally, all large peptide sequences generated from potentially cross-reactive proteins (positive hits) were collected and run against the AllerCatPro 2.0 database. This second search was made to assess the similarity between peptides and known allergens (number of 3x6-mers and percentage identity to 3d epitope), i.e. to what extent the sequences that were suspected of causing protein cross-reactivity, were maintained in the peptides after digestive cleavage.

## 3 Results

### 3.1 SDS-PAGE

**Fig. 1:**
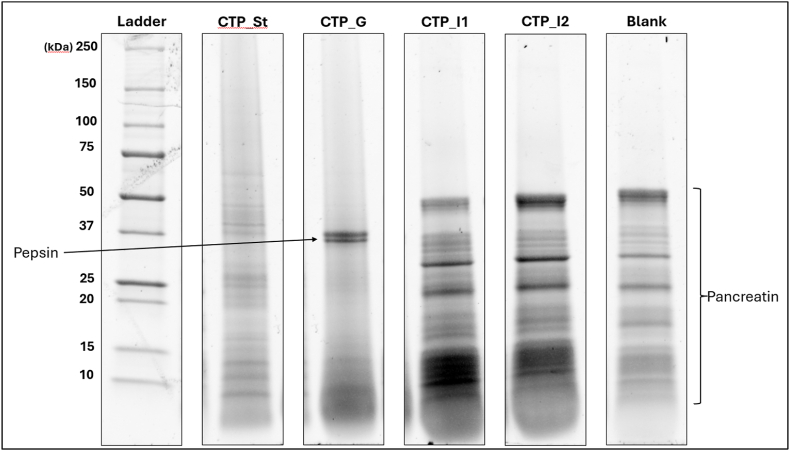
Non-reducing SDS-PAGE protein profiles of digestion samples. Each column represents one analyzed sample (Ladder – protein standard; CTP_St – stability (food & no enzymes); CTP_G – gastric phase; CTP_I1 - 1-h intestinal phase; CTP_I2 - 2-h intestinal phase; Blank – enzymes & no food). The bands introduced by the added digestive agents (pepsin and pancreatin) are indicated.

The SDS-PAGE results, shown in Fig. 1, presented the digestibility profile of the Clostridium tyrobutyricum powder (CTP). The stability sample (CTP_St) showed the protein bands introduced by the sample itself and their resistance to the digestive conditions in the absence of enzymes. It can be clearly observed that a wide variety of proteins were present in the stability sample, with weights ranging from 150 kDa to 10 kDa and lower. Samples collected after the 2-h gastric phase (CTP_G) indicated extensive protein breakdown due to pepsin proteolytic activity, with very few of the sample bands remaining, all below 15 kDa. Samples collected from the intestinal phase, after one (CTP_I1) and two (CTP_I2) hours of digestion showed further degradation of the peptides, demonstrated by the larger area below 10 kDa that appeared colored. Finally, to ensure the reliability of these observations, bands introduced by the digestive enzymes, shown as “Blank”, could be compared to the test samples.

### 3.2 Peptide distribution and persistence

**Fig. 2:**
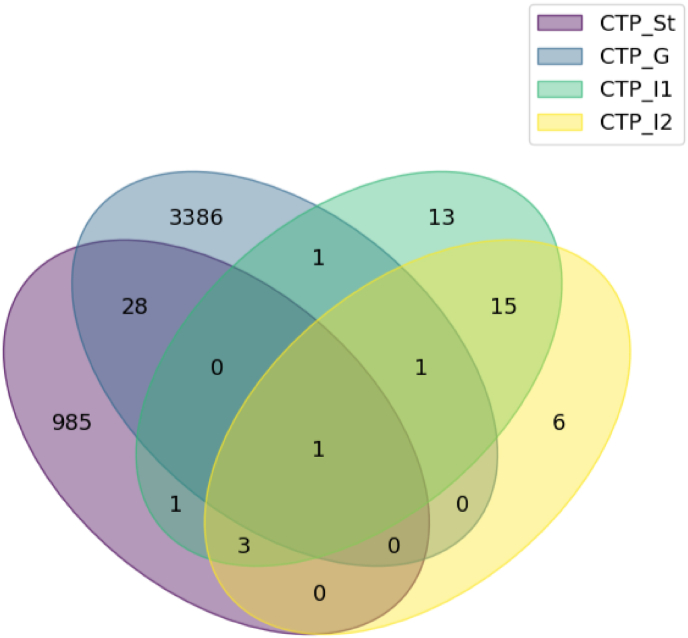
Venn diagram of unique peptide sequence distribution among *in vitro* digestion samples. Each ellipse represents one analyzed sample (purple for stability, CTP_St; blue for gastric phase, CTP_G; green for 1-h intestinal phase, CTP_I1; yellow for 2-h intestinal phase, CTP_I2). The sum of the numbers within each ellipse represents the total number of unique peptide sequences present in the sample, while the number in the overlap of a given set of ellipses represents the number of unique peptide sequences that appear in multiple samples. Numbers that appear within one ellipse alone (outside of the overlap) represent the number of unique peptide sequences that were detected in this one sample alone.

To obtain a clearer understanding of the generated peptides that appeared below the 10 kDa mark on the SDS-PAGE gels, the number of generated peptides between 9 and 80aa in length and their distribution among different phases were visualized in a Venn diagram (Fig. 2). It could be observed that the stability sample shows many (1018) unique peptide sequences, which were generated for proteolysis due to the digestive conditions alone, while plenty of molecules longer than 80aa still remained in this sample, as described in the previous section. After observation of the gastric phase, it was clear that a substantial number (3417) of 9aa or longer peptides can survive gastric digestion, while only a few unique sequences remained after 1 h (35) and 2 h (26) of intestinal digestion. This analysis provided deep insight into the digestibility of the product and could act as a screening step, identifying persistent molecules that survive multiple digestive phases, as targets of interest for both IgE and non-IgE allergenicity assessment.

When comparing the samples, it is interesting to see how many peptides appear in multiple phases, suggesting structural resistance to a particular enzyme or set of conditions. For example, twenty-nine (29) peptide sequences appeared in both CTP_St and CTP_G, which can lead to the assumption that these sequences are generated through the change of conditions and were somewhat resistant to further proteolysis by pepsin. Additionally, comparing the two different timepoints of the intestinal phase, CTP_I1 and CTP_I2, provided information about the molecular stability of peptides in the intestine. It was evident that from the thousands of peptides that survived gastric digestion, only a few (35) maintained a length of 9 or more aa after 1 h of intestinal digestion, and even fewer (26) after 2 h. Nine (9) peptides survived intestinal digestion for 1 h but not two. This information could be valuable in distinguishing transient from persistent peptides in the intestinal phase and can easily be obtained from other phases if required. Furthermore, the presence of one (1) peptide sequence in all four phases suggested that it remained partly unaffected by enzymic action. Several observations can be made by examining the overlaps in this diagram, but it is important to note that it only paints a qualitative picture of peptide distribution, without considering peptide abundance.

### 3.3 Large peptide distribution and abundance

**Fig. 3:**
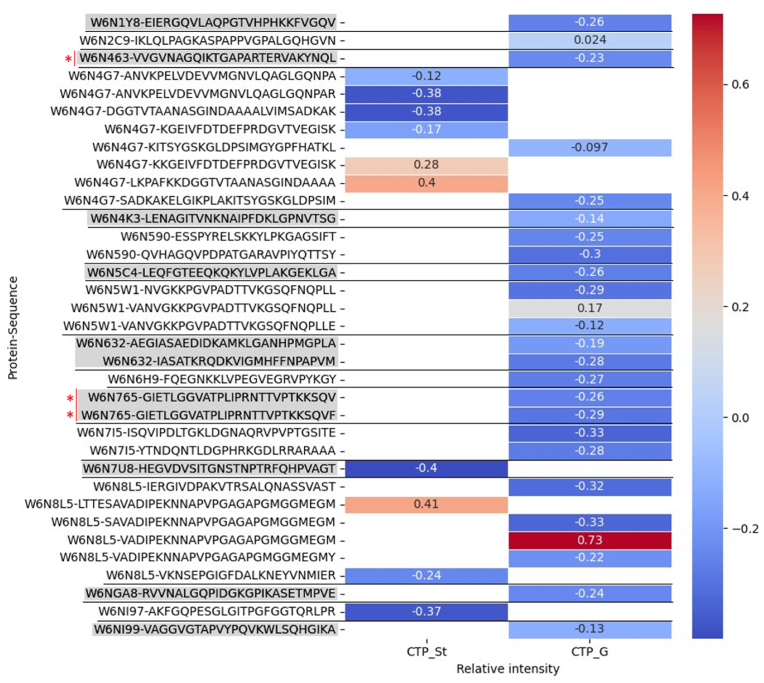
Heatmap of large (*≥*2450 Da) peptide distribution with z-score values of relative intensity. Deep blue indicates minimal intensity while deep red indicates maximal intensity, among the large peptide values. On the y-axis exact peptide sequences are shown along with the names of their predicted parent proteins and on the x-axis the different digestion samples are shown with the map values showing the z-scores of relative peptide intensities within each sample (CTP_St – stability (food & no enzymes); CTP_G – gastric phase). No large peptides appeared in all three replicates of CTP_I1 (1-h intestinal phase) and CTP_I2 (2-h intestinal phase), hence these columns have been excluded. The alternating patern of grey and blank entry background is used to indicate peptides that originate from the same protein. Red side bars and asterisks indicate peptides flagged as positive hits in the online database cross-reactivity search.

Large peptides (≥2.45 kDa) have a higher capacity to elicit an allergic reaction (Mills et al., 2024). Their distribution was visualized in a heatmap, shown in Fig. 3, demonstrating abundance among large peptides and also presenting the relative intensity z-score values, calculated from all the peptides present in each sample. Additionally, the code of the predicted parent protein was shown next to the peptide sequence, which assisted in identifying peptides with small aa differences. The two intestinal phase samples were excluded from the heatmap, as they showed no large peptide sequences in all three replicates.

The CTP_St column was included as a standard of comparison, to potentially detect protein structures that remained largely unaffected by proteolytic cleavage in the stomach. One such sequence was identified, occurring from protein W6N8L5, which appeared in CTP_St in high abundance (0.41) as part of a 32aa peptide and was also highly abundant (0.73) in CTP_G as a 26aa peptide, suggesting that the major effect of gastric digestion was cleavage of 6 aa off the starting position. Nevertheless, this was not studied further, as W6N8L5 showed no evidence of cross reactivity with known allergens.

The predicted parent protein entries show that out of all the proteins (707) present in the biomass, fifteen proteins (shown in Table 1) generated large peptides (in total 35 large peptides were identified) after gastric digestion. Also, when examining the CTP_G column of the heatmap, it became clear that most large peptides showed abundance lower than the mean in the gastric phase, with only three entries showing positive z-scores.

**Table 1:** Results of protein sequence identity search with two online tools.

Protein	Length	Best known allergen hit name	Species	Source	Identity linear 80aa window
W6N632	282	No significant hit	–	–	–
W6N4G7	393	No significant hit	–	–	–
W6N1Y8	397	No significant hit	–	–	–
W6N590	427	No significant hit	–	–	–
W6N6H9	484	No significant hit	–	–	–
W6N765	617	Aed a 8	*Aedes aegypti*	AllerCatPro, AllergenOnline	73.8 %
W6N8L5	545	No significant hit	–	–	–
W6N2C9	141	No significant hit	–	–	–
W6N7I5	334	Per a 13	*Periplaneta americana*	AllerCatPro, AllergenOnline	61.2–61.7 %
W6N4K3	411	No significant hit	–	–	–
W6N5C4	379	No significant hit	–	–	–
W6N5W1	1181	Poa p 9	*Poa pratensis*	AllergenOnline	36.26 %
W6NGA8	504	No significant hit	–	–	–
W6NI99	296	No significant hit	–	–	–
W6N463	431	Sal s 2	*Salmo salar*	AllerCatPro, AllergenOnline	68.8–70.01 %

### 3.4 Parent protein allergenicity assessment

Protein sequences were obtained from UniProt for all the parent proteins that generate large peptides resistant to gastric digestion *in vitro*. The sequences were investigated for sequence identity to known allergens higher than 35 % over an 80aa window, using two online tools, AllerCatPro 2.0 and AllergenOnline. The results of this search were summarized in Table 1. Out of the fifteen protein sequences investigated, only four returned positive hits (W6N765, W6N7I5, W6N5W1 and W6N463). With the use of online databases all the positive hits were identified, though only the best hit for each protein was shown. Moreover, the organism species as well as the percentage of linear identity over an 80aa window is shown. The databases were not always in agreement regarding the best hit or the identity percentage; hence, it was evident that the choice of database can influence the result of the search.

### 3.5 Peptide cross-reactivity assessment

Finally, as a last step in the analysis, the presence of the predicted cross-reactive epitope in the generated peptides was examined. The peptide sequences generated from the four potentially cross-reactive proteins were selected and searched against the AllerCatPro 2.0 database to evaluate two key metrics, the number of allergens that shared at least 3 hexamers with the query peptide and the percentage of identity to the 3D predicted allergen epitope. The comparison of 3 hexamer overlaps utilizes a robust criterion, where the sliding 80aa window comparison was not applicable, and offered valuable information on the number of known allergens with which a peptide might show potential cross reactivity, while the percentage of identity to the 3D epitope offered qualitative information on the similarity between the peptide and the best hit. This database could prove to be an invaluable tool in allergenicity assessment, as it uses epitope mapping to predict 3-dimensional epitopes of known allergens (Maurer-Stroh et al., 2019), to which the digested peptides can be compared. It is shown, on Table 2, that only peptides that originated from two of the four parent proteins generated positive hits, maintaining some similarity to predicted allergen epitopes after cleavage. As expected, peptides occurring from protein W6N5W1, which only generated positive hits through AllergenOnline, were not picked up as potentially allergenic by AllerCatPro. Instead, a full FASTA search of the peptide sequences on AllergenOnline did not result in any positive matches. Predictably, the best hit for the peptides is different than what it was for the entire proteins but the similarity of the remaining sequence to known allergens can be useful in determining the need for follow-up studies *in vitro*.

**Table 2:** Results of peptide-3D epitope identity search with AllerCatPro 2.0.

Protein	Generated peptide (position)	Best known allergen hit name	Species	# of 3x6-mer overlaps	% identity 3D epitope	Relative intensity (z-score)
W6N765	372–397	Unknown	*Oryza sativa*	2	52.6	−0.259566
W6N765	372–398	Cor a 10	*Corylus avellana*	2	52.6	−0.292796
W6N5W1	788–811	No significant hit	–	0	–	−0.294418
W6N5W1	786–811	No significant hit	–	0	–	0.167606
W6N5W1	786–812	No significant hit	–	0	–	−0.124071
W6N463	382–407	Gal d 9	*Gallus gallus*	32	38.5	−0.233235
W6N7I5	179–202	No significant hit	–	0	–	−0.276097
W6N7I5	216–243	No significant hit	–	0	–	−0.333675

## 4 Discussion

### 4.1 Results interpretation

The present study aimed to develop a refined screening strategy for the identification of proteins from complex food mixtures (such as SCPs) that are of potential concern for IgE-mediated allergenicity by employing a combination of *in vitro* digestion and peptidomics methods. It is, to the best of our knowledge, the only such approach presented to date and it answers the call for better integration of *in vitro* testing and modernization of *in silico* tools with more targeted databases, as made by the EFSA NDA Panel, in 2022 (Mullins et al., 2022). Strategies to integrate *in vitro* digestion into NF allergenicity assessment have been developed by the European Food Risk Assessment program and published by EFSA (Garciarena et al., 2022; Liguori et al., 2022) but they focus on immunoassays using digestates, rather than proteomics analysis and all the related results presented here. The integration of proteomics into the search for allergenic properties in NFs has also generated some interest lately, best showcased by the work of Bianco et al. (2022), though that approach does not include *in vitro* digestion, which can be valuable for exposure assessment to novel allergens. Perhaps the strategy most similar to our own was presented by Di Stasio et al. (2017), though that study was targeted for peanut allergens, while our strategy aims to serve as a non-targeted filtering step in NF risk assessment.

The interpretation of results obtained by novel allergenicity metrics can be challenging, due to the lack of experience with the method and establishing real world outcomes. As such, using novel methods should be done with great care to avoid making inappropriate conclusions. The current approach is exploratory rather than confirmatory; therefore, the findings from this analysis offer probabilistic insights into the likelihood of peptide–immune system interactions that may indicate allergenic potential, based on digested peptide size, persistence, and abundance, as suggested by Fernandez et al. (2019). Allergenicity assessment by the means of probabilistic modeling is a strategy already presented by an EFSA-funded project in 2019 (German Federal Institute for Risk Assessment et al., 2019). As EFSA takes step towards modernizing the existing frameworks with NGRA tools and Findable Accessible Interoperable and Reusable (FAIR) data management practices (Pineda-Pampliega et al., 2022), it is perhaps beneficial to develop workflows that can produce a wide range of data in relation to allergen stability and abundance, which could assist with exposure assessment. Though interpreting the readouts of *in vitro* digestibility tests might be yet unclear, along with the potential relationship between digestibility and allergenicity (Mills et al., 2024), this novel approach would provide a “piece of the puzzle” within the totality of available evidence in assessing the allergenicity potential of novel food ingredients.

Initially, when examining the SDS-PAGE results and the peptide distribution Venn diagram, it is evident that CTP is a highly digestible food with very few peptides larger than 8aa in length surviving digestion *in vitro*. After the end of the digestive process, twenty-six unique peptides remain (larger than 8aa in length). In theory, these peptides could have the greatest relative concern out of all the peptides in the sample, as they may have greater time to be taken up for professional or non-professional antigen presentation in the intestinal tissue and therefore could represent the constituents to be studied further to determine whether their sequence and abundance pose a threat for potential IgE or non-IgE immunogenicity. In the same spirit, peptides that appear after 1 h of intestinal digestion but not after two, show lower relative concern as they could remain in the intestine for considerable time but will be expected to not survive as long, and peptides that appear at the end of the gastric phase but not in any of the intestinal phases show even lower concern and so forth.

As this study focused primarily on IgE cross-reactivity, further analysis on peptide sequence and abundance was conducted only for peptides of weight heavier than or equal to 2.45 kDa. By studying Fig. 3, we see that, based on the negative z-scores of most large peptides, the most abundant peptides occurring from *in vitro* digestion of CTP are of mass lower than 2.45 kDa. This is an initial indication of low likelihood of eliciting IgE-mediated allergic reactions, judging by measures of size, persistence and abundance, since large peptides comprise about 1 % of the total number of peptides that survive gastric digestion (35/3417), and only three of those show abundance higher than the mean (less than 0.1 %). Furthermore, no large peptides survive 1 h of intestinal digestion. Nevertheless, since these measures are only indicative of concern based on persistence and abundance, cross-reactivity analysis of the peptide sequences was conducted to further evaluate potential for allergenicity.

Taken together, the peptide distribution Venn diagram and the large peptide distribution heatmap clearly indicate an inverse correlation between peptide size and duration of the digestion, as can be expected in digestible foods. As the originally abundant proteins are cleaved, they generate a larger number of unique peptide sequences (3417 in CTP_G), each with reduced relative intensity. The longer the digestion lasts, the fewer unique peptide sequences appear (35 in CTP_I1, 26 in CTP_I2), and the peptide chain length is reduced due to enzymic cleavage. The assessment of peptide abundance can assist in identifying persistent peptides, as they would show high z-scores and large chain length over time, deviating from the expected pattern. Grouping peptides based on parent protein, as shown in Fig. 3, can reliably detect structures that are resistant to digestion and require more scrutiny during risk assessment.

Even though large peptide abundance was assessed in the previous step, there is no clear cutoff beyond which peptides would be eliminated from further analysis. Therefore, the parent proteins of all large peptides were scanned for cross-reactivity, as shown in Table 1. In this step, risk assessors can evaluate the positive hits in combination with other information such as allergen prevalence, strength of evidence, sequence identity percentage and decide whether further *in vitro* assessment is needed (Mills et al., 2024). Here, positive hits themselves are not evaluated, but presented as a guide for further analysis, studying whether peptides generated from positive hit proteins maintain similarity to the predicted allergen epitope after digestive cleavage. These results offer an additional layer of information, analyzing potential cross-reactivity of digested peptides rather than original proteins. As shown in Tables 2 and it is evident that out of all the proteins present in the complex mixture that was digested *in vitro*, only two unique peptidic structures (three peptide entries appear on the table but two of them come from the same protein and only differ by a single aa) maintain large size and medium level of structural similarity to a predicted 3D epitope of a known allergen at the end of gastric digestion. Additionally, all three peptides show abundance lower than the mean (negative z-score). Collectively, these results suggest a low likelihood of allergic reaction due to cross reactivity from CTP, based on peptide size, persistence and abundance. Nonetheless, it is important to mention that this information alone does not suffice for a complete allergenicity assessment of NFs, and confirmatory studies (e.g., IgE binding tests) could be necessary to express a robust conclusion to the assessment. Also, predicted epitopes can introduce uncertainty into the assessment. It can be easily understood that this last step of analysis should perform better for well-known and characterized allergen epitopes.

### 4.2 Assessment strategy

#### 4.2.1 Theoretical basis

The bulk of protein digestion begins in the stomach where pepsin facilitates the hydrolysis of peptide bonds at optimal pH values between 2 and 3, showing wide enzyme specificity (Bøgh and Madsen, 2016). The main role of the stomach is to store ingested food, break down large solids (mechanically) and nutrients (chemically), and gradually deliver it to the small intestine. The rate of delivery (rate of gastric emptying) depends on several factors, among which is the nature of the meal (Hellström et al., 2006). Generally, gastric emptying occurs faster for liquids than solids, and, theoretically, a small percentage of any food can pass through to the intestine within the first few minutes after ingestion. This, in combination with the fact that allergic reactions can occur at very small doses of the allergen, makes it a possibility that food allergens can reach their target site without facing major breakdown, maintaining much of their structural integrity. It could, in theory, explain the fact that proteins which are known to be labile in the conditions of the GI tract can still elicit severe allergic reactions.

Even though symptoms in the mouth (e.g., oral allergy syndrome) have been known to occur in certain cases, it is widely accepted that the major mechanisms leading to elicitation revolve around antigen uptake and presentation by the immune cells of the lamina propria after complex interplay of several cells that reside in the small intestine (Vickery et al., 2011; Samadi et al., 2018). The allergic reaction progresses with allergen binding the specific IgE antibody on the surface of the mast cell, leading to degranulation (Vickery et al., 2011; Valenta et al., 2015). Therefore, it is safe to assume that any allergenic molecule, up until the point of binding, must obtain and maintain a size large enough to bind to its respective IgE antibody.

The effect of digestibility on the allergenic potential of a protein has been an open topic of debate between scientists for at least three decades since Astwood et al. (1996) first studied the resistance of known food allergens to pepsin digestion. In the following years, as the interest in this effect grew, so did the conflicting reports, failing to establish a clear relationship between digestibility and allergenicity, as it was comprehensibly reviewed by Bøgh and Madsen (2016) and then existing gaps in this relationship were identified, such as the roles of endolysosomal digestion and the gut microbiota, by the shared work of two COST action groups (Verhoeckx et al., 2019). To this day, allergenicity assessment and the role of digestibility remain complex topics of discussion among EFSA (Mills et al., 2024) and stakeholders and can sometimes cause confusion to companies aiming to apply for NF authorization in the EU.

#### 4.2.2 Strategy development

In alignment with Directive 2010/63/EU on the protection of animals used for scientific purposes and our commitment to NGRA, we employed exclusively non-animal methods. However, the use of animal-derived products remained unavoidable. Our strategy focuses on obtaining accurate information at protein level from the sample, while offering adjustability to specific research needs and the ability for automation or standardization of the conditions where applicable. Thus, the methods were selected with the potential for quick and robust interlaboratory use in mind.

When developing this strategy, the main objective of the study was obtaining valuable information regarding protein cross reactivity that could lead to IgE-mediated immune responses. Nevertheless, the same set of analyses can be used for acquiring information regarding non-IgE allergenicity. Digestion of protein samples *in vitro* is a clear first step for the assessment, as it is a process possibly as crucial as metabolism is in small-molecule toxicology, because it allows for the study of the final peptide form, rather than the original protein. Currently, no gold standard measurements exist to assess the allergenic potential of proteins or peptides. However, based on EFSA recommendations, the most critical factors appear to be peptide size, sequence identity to known allergens, persistence and abundance of peptides after digestion (Naegeli et al., 2017; Fernandez et al., 2019). While a clear peptide length cutoff of 9 aa has been suggested for non-IgE mediated immune responses, below which the immunogenic potential can be considered to be low (Naegeli et al., 2017), there is no clear consensus on a cutoff value for IgE-mediated responses. Considering the most recent EFSA document, which suggests that protein “hydrolysates with a size distribution of less than 2.5 kDa have a much-reduced capacity to elicit allergic reactions” (Mills et al., 2024) and the fact that potential endolysosomal degradation of allergens cannot be easily accounted for, it becomes apparent that the best option is to first filter the digested peptides based on their size (and weight). Several cutoffs can be applied (e.g., weight ≥2.45 kDa, length > 8aa) before subsequent analysis of peptide sequence, persistence and abundance, to address the most relevant endpoint.

**Fig. 4:**
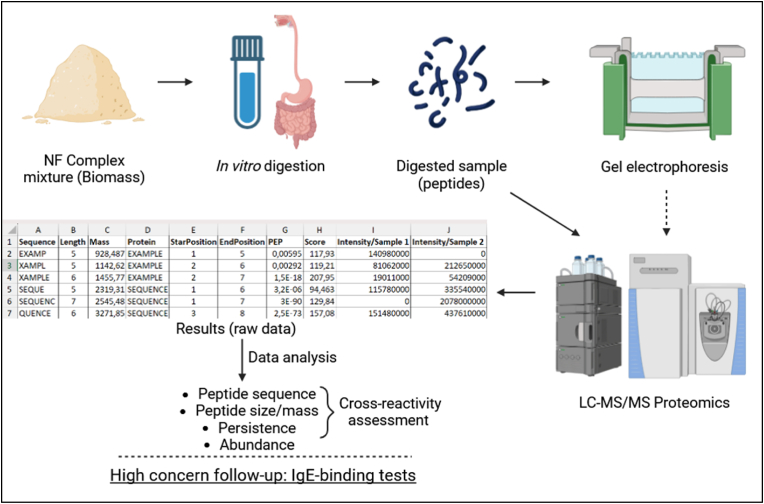
Schematic visualization of the assessment strategy.

As mentioned, notable knowledge gaps in the field have already been identified (Verhoeckx et al., 2019). Several of these gaps (e.g., pH, enzyme/protein ratio) concern assay conditions. Standardizing the digestion conditions is of paramount importance to succeed in reproducible qualitative assessment of peptide generation. Also important are the conditions of sample storage, treatment and analysis that could affect sample behavior. In our approach, the internationally standardized *in vitro* digestion protocol INFOGEST (Brodkorb et al., 2019) was used, and further treatment prioritized keeping sample interference at a minimum. In this way, specific parameters, such as enzyme/protein ratio, can be adjusted to specific research needs (e.g., anticipated daily intake, or intake per meal) while maintaining the rest of the conditions stable and thus generating comparable results. Analysis by SDS-PAGE allows for reproducible peptide detection of a wide range of weights (from a few hundred to a few kDa). However, this is not the complete picture, as peptide identification is not possible, and peptides of lower weight that could still be allergenic cannot be detected. Therefore, further analysis can be conducted using mass spectrometry-based proteomics. The SDS-PAGE results (along with other sample-specific factors) can serve as a guide to deciding if further analysis is needed and which proteomics method is most suitable for the sample in question. For example, the presence of large persistent peptides or intact proteins may require top-down proteomics, while focusing on the smaller peptides (<5 kDa), as was the case with this study, warrants the use of bottom-up proteomics. Finally, the major readouts of the method are the peptide sequences along with specific characteristics, such as length and exact mass, and their respective confidence scores that can act as filters to further strengthen the quality of the results. The resulting dataset is a virtual goldmine of information which can be used not only in allergenicity assessment, but also in digestibility studies, since the bulk of reliable *in vitro* proteolytic cleavage information could be utilized to boost *in silico* digestion modeling (Fig. 4).

#### 4.2.3 Output and limitations

The state of the starting material and the digestion conditions play a major role in determining the peptide content of the generated samples. *In vitro* digestion allows for sampling at multiple time points, thus acquiring kinetic information of the digestive process (Minekus et al., 2014). The peptide content of the samples, visualized through SDS-PAGE, gives an initial idea of the size of persistent and abundant peptides, though these results alone describe product digestibility and have little value for allergenicity assessment. As mentioned, one of the biggest advantages of proteomics (or peptidomics) methods is that they can generate large datasets that can be analyzed in multiple ways, offering plenty of options to researchers (or risk assessors) to study different endpoints. After applying a confidence score filter based on study objectives, the major readouts can be compared between different digestion timepoints of a single sample (kinetic measurements), between samples at the same timepoint (putative allergens vs reference protein), while also possible are size distribution analyses during each digestive phase (e.g., number of unique peptides heavier than 2.5 kDa that survive gastric digestion) and specific sequence monitoring throughout the digestive process. These examples clearly show the flexibility of the method, and there are probably other analyses to be made that were not considered within our study, which only strengthens the case for the use of Omics approaches in risk assessment. While lacking appraisal of a clear allergenicity endpoint, this combination of methods offers an array of valuable information which the risk assessors can examine on a case-by-case basis (selecting the most relevant information for each product) and incorporate in the weight-of-evidence approach, perfectly aligned with the current regulatory framework (Naegeli et al., 2017; Fytsilis et al., 2024; Turck et al., 2024).

**Fig. 5:**
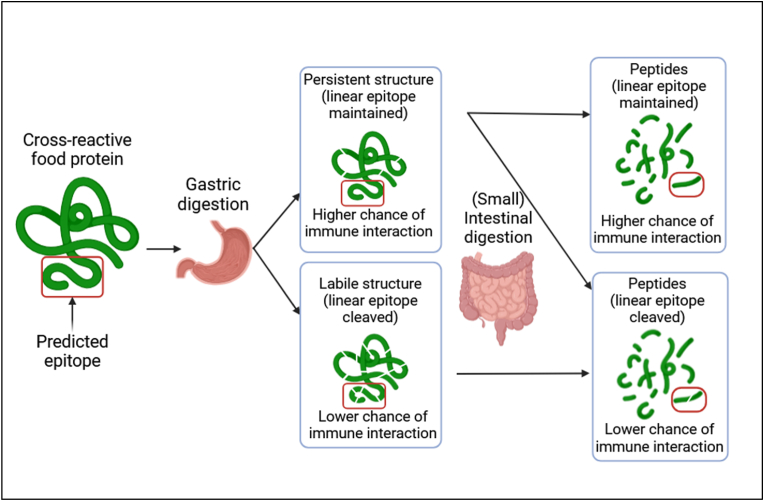
Schematic visualization of all possible scenarios regarding the fate of cross-reactive food protein epitope in the GI tract, along with the likelihood of interacting with the immune system and eliciting an allergic reaction.

Incorporating information from NGRA tools (or any novel tools) into allergenicity assessment can be expected to introduce non-standard uncertainties, that would need to be analyzed on a case-by-case basis (Benford et al., 2018), since these tools are not part of the current standardized approach. However, the novelty of the method is accompanied by limited knowledge valorization. What has been made clear is how these new tools can be used to obtain deep insight into the stability, abundance and general transience of potential allergens in the GI tract. This insight pertains to the relative amount of time a peptide will pass in the small intestine, where it could potentially interact with the immune system. Therefore, the exploratory nature of our findings permits only probabilistic interpretation regarding a protein fragment's potential to elicit an allergic response and precludes definitive conclusions. This is why we believe that these results should be considered along with other relevant information in the allergenicity assessment process, probably as part of tier II (Turck et al., 2024). The rationale for the interpretation of the results has been schematically visualized in Fig. 5, which is only indicative of the idea behind our approach, without accounting for all allergenicity-relevant aspects (e.g., 3D epitopes).

As is usually the case with most methods, this strategy also has several limitations. Analyses of complex food mixtures, such as SCPs, are very complicated due to the sheer number of different nutrients involved and sample heterogeneity, without even considering batch-to-batch variations (Barthélémy et al., 2023). Even though raw datasets typically contain tens of thousands of peptide entries, data processing can be automated to create a seamless workflow. Additionally, the INFOGEST protocol is a static *in vitro* digestion model, which fails to mimic important *in vivo* processes such as gastric emptying, dynamic pH changes or microbiome presence (Mackie & Rigby, InfoGest Consensus Method). Nonetheless, it still offers the best (to our knowledge) option for a benchtop standardized *in vitro* digestion model, which is simple and affordable to set up without the need for specialized lab equipment. Dynamic models, like the TNO gastrointestinal model (TIM) would offer a significantly better replication of *in vivo* conditions, but would also significantly raise the overall cost, time and labor required (Minekus, The TNO Gastro-Intestinal Model). While the assessment of allergenic potential based on peptide persistence and abundance is a promising strategy, it is important to mention that there is no clear correlation between the metrics and allergenic potential as of yet. Reviewing the available literature did not result in a clear link between allergenicity and digestibility (Bøgh and Madsen, 2016) and the research gaps that need to be addressed in the field have been extensively discussed in a recent work (Verhoeckx et al., 2019). However, obtaining data under standardized conditions and through highly accurate techniques, as presented, along with defining criteria to identify stable, persistent and abundant peptide fragments after digestion (Casacuberta et al., 2025) should elucidate any potential links in the future. Moreover, as previously mentioned, the selection of the online database, as well as the predefined search criteria can influence the results of the cross-reactivity search and would need to be refined as EFSA moved towards more advanced *in silico* approaches (Casacuberta et al., 2025). The performance of the most relevant databases and search criteria and how they might affect the outcome of the assessment have been the subject of multiple studies and discussions in recent years (Abdelmoteleb et al., 2021; Mills et al., 2024; Casacuberta et al., 2025). Finally, the complete assessment of allergenic potential must account for the molecular properties of type I allergens: the ability to sensitize and individual, the ability to elicit an allergic reaction and the ability to bind to IgE antibodies. None of these properties can be verifiably assessed through our strategy, which is why it is important to apply the aforementioned methods as a complementary measure to the current framework, to create an improved screening step for the allergenicity assessment of novel foods.

### 4.3 Current challenges and future perspectives

In the past decade several calls have been made by researchers and risk assessors alike for the integration of more advanced research tools into food risk assessment (Fernandez et al., 2019, 2021; Mullins et al., 2022). Here, we have explored the options offered by integrating *in vitro* digestion and peptidomics into allergenicity assessment of a single cell protein. In our view, this strategy can accurately and reliably deal with elucidating the fate of complex protein mixtures such as SCPs in the GI tract.

The case-by-case basis of NF risk assessment can limit the stakeholders’ understanding of different criteria and their weight in the evaluation of a product (de Boer and Bast, 2018; Fytsilis et al., 2024). There is a clear need for better insight into the digestion of complex mixtures, because of their convoluted nature that significantly complicates the assessment process. For instance, a full qualitative search of all the protein sequences present in a microbial biomass can naturally result in hundreds or thousands of unique molecules, with several of them showing high identity to known allergens, often without posing any actual risk (Abdelmoteleb et al., 2021). It would be costly and impractical to test all positive hits for IgE-binding capacity, and sera of well-characterized allergic individuals are not always available. Therefore, there is a need for accurate and sensitive screening steps between the genome/proteome and phylogenetic assessment of a food ingredient and the *in vitro* testing that follows positive cross-reactivity results. Our approach is based on a simple *in vitro* digestion – peptidomics workflow which offers deep insight into product digestibility and stable fragment size, persistence and abundance, providing risk-assessors with valuable information towards identifying the most relevant peptides that could require testing *in vitro*. Moreover, it could also assist stakeholders with the comprehension of the assessment process and potentially reduce the overall time to reach a decision, in this way facilitating food innovation in the EU.

The current guidance for complex mixture NF authorization already requires applicants to submit studies on product digestibility *in vitro* to assess the nutritional value of the ingredient as a protein source, and complete at least the first tier of allergenicity assessment (comprehensive literature review and phylogenetic studies) while completion of the following tiers is case-by-case requested based on Tier I results (Turck et al., 2024). This is a logical screening step before any further analysis is conducted. Depending on the findings of this Tier I search, the second Tier is triggered in which cross-reactivity is predicted by comparing the protein amino acid sequence (AAS) homology to results in allergen-sequence databases (e.g., FASTA local alignment algorithm or the Basic Local Alignment Search Algorithm (BLAST)) with a default threshold of 35 % identity over at least 80 amino acids. Although noted by EFSA as being highly conservative (i.e., too many false positives), this approach provides a transparent and reproducible evaluation that has been frequently utilized by industry and government risk assessors. If the results of bioinformatic analyses (Tier II) suggest potential cross-allergenicity with a known food allergen that triggers severe IgE-mediated allergic reactions in sensitive individuals, further analysis of specific IgE binding using human serum *in vitro* would be performed (Tier III). Finally, positive results in Tier III analysis would trigger Tier IV, consisting of skin-prick tests and an oral food challenge in subjects with confirmed food allergy to the known allergen. Not captured within this data set, is an assessment of the stability of the novel protein in the GI tract, for example, more specifically, the effect of digestion of the proteins into low molecular weight peptides, and subsequent impact on allergenicity.

The approach we present in this paper perfectly aligns with the current framework. Methods to assess protein digestibility *in vitro*, such as the INFOGEST protocol, follow standardized conditions that include physiologically relevant steps such as the addition of bile in the intestinal phase, which are important for improved prediction of mixture behavior in the GI tract (Maldonado-Valderrama et al., 2011). After phylogenetic studies, samples generated from the digestive process can be conditionally evaluated with electrophoresis and, on a case-by-case basis, with proteomics analyses as part of Tier II testing, while the results can be processed in automated fashion, using open-source programming tools like R and Python. Finally, after this refined filtering step, proteins and peptides of interest can still be assessed for IgE-binding if deemed necessary (Tier III). Overall, this approach allows risk assessors to obtain a better understanding of the product in small, tiered analytical steps that may allow for in-between decisions on whether further assessment is required. Additionally, they can utilize readouts and cutoffs to integrate the most relevant information for each product into the weight-of-evidence approach. Meanwhile, applicants acquire a clear picture of the fate of the product in the GI tract, that could potentially help with product design or health claims and face reduced uncertainty over the assessment process, reducing the need to employ several additional analyses.

One of the main advantages offered by this approach is the flexibility to be applied to any product, with or without prior phylogenetic studies, and the ability to fine-tune it to fit any particular research questions. Through *in vitro* digestion arises the opportunity to easily amend the risk assessment based on the different consumer target groups. For example, foods destined for consumption by children (e.g., infant formulas), or the elderly can be assessed based on the presumed digestive conditions of the specific target group instead of those of the general population (Minekus et al., 2014; Gold et al., 2020). Additionally, our approach allows for the monitoring of processed foods rather than original proteins only. If a food has a specific anticipated use (e.g., frying) then it can be digested after processing and analyzed through glycopeptidomics to examine the glycosylation levels and other structural modifications relevant to risk assessment (Koh et al., 2022; Hale et al., 2024). This method essentially creates a pipeline for generating digestibility information that could be used beyond assessment of product safety, to understand allergen characteristics a lot better and possibly develop more advanced risk assessment tools.

Nonetheless, NF allergenicity prediction remains a complicated matter. The current method takes a stepwise approach based on key factors that are relevant to the allergenic potential of an ingredient. However, several methods that are part of the current framework show limitations in various aspects, and several knowledge gaps remain unaddressed (Verhoeckx et al., 2019; Fernandez et al., 2024). In general, it is a challenging task for any toxicological method to prove or correctly predict product safety towards everyone, and it becomes even more challenging when the product in question is of a highly complex nature. A realistic goal of current risk assessment would be to create a comprehensive spectrum of likelihood for protein allergenicity towards the general population or specific cohorts, while striving to develop and integrate more accurate and relevant tools. To achieve this, better understanding of allergenicity regarding structural characteristics of food allergens, epitopes and sensitization potential is needed. Ultimately, our findings strengthen our belief that improving the current framework through the implementation of novel and accurate tools in NF risk assessment can significantly contribute to illuminating the field and filling the existing knowledge gaps.

## CRediT statement

Vaios D. Fytsilis: Conceptualization, Methodology, Software, Formal analysis, Investigation, Writing - Original Draft, Visualization.

Rensong Ji: Software, Investigation, Writing – Review & Editing.

Karli R. Reiding: Investigation, Writing – Review & Editing.

Albert J.R. Heck: Investigation, Writing – Review & Editing.

Frederik-Jan van Schooten: Conceptualization, Supervision, Writing – Review & Editing.

Alie de Boer: Conceptualization, Supervision, Writing – Review & Editing, Funding acquisition.

Misha F. Vrolijk: Conceptualization, Supervision, Writing – Review & Editing, Funding acquisition.

## Declaration of competing interest

None.
